# Transcultural Adaptation and Piloting of the “Regarding Blood-Sampling Practices at Primary Health Care Centres” Questionnaire

**DOI:** 10.3390/ijerph17103541

**Published:** 2020-05-19

**Authors:** Adolfo Romero-Arana, Blanca Prieto-Callejero, Javier Fagundo-Rivera, Juan Gómez-Salgado, Macarena Romero-Martín, Carlos Ruiz-Frutos, Adolfo Romero

**Affiliations:** 1 Andalusian Public Foundation for the Biomedical Research in Malaga (FIMABIS), Nursing Intensive Care Unit, Hospital Regional Universitario, 29010 Málaga, Spain; adolfo9381@gmail.com; 2 Hospital Virgen de la Bella. Lepe, 21440 Huelva, Spain; blanca.prietoc@gmail.com; 3 Andalusian Health Service, Health Sciences Doctorate School, University of Huelva, 21007 Huelva, Spain; javier.fagundo308@alu.uhu.es; 4 Faculty of Social Work, Department of Sociology, Social Work and Public Health, University of Huelva, 21007 Huelva, Spain; frutos@uhu.es; 5 Safety and Health Postgraduate Programme, Universidad Espíritu Santo, Guayaquil 091650, Ecuador; 6 Faculty of Nursing, Department of Nursing, University of Huelva, 21071 Huelva, Spain; 7 Instituto de Investigación Biomédica de Málaga (IBIMA), Nursing and Podiatry Department, Health Sciences School, University of Málaga, 29010 Málaga, Spain; preanalitica@gmail.com

**Keywords:** nursing methodology research, preanalytical errors, blood sampling, nursing, primary care, transcultural adaptation

## Abstract

Preanalytical errors account for up to 70% of the total potentially detectable errors in the laboratory. The main problems detected are related with procedures associated with Primary Care nursing practices that are directly involved in the preanalytical phase. The objective of this study is to carry out a transcultural adaptation and piloting of the “Regarding Blood-Sampling Practices at Primary Health Care Centres” questionnaire as regards blood sampling in Primary Care. For this, a cross-sectional descriptive study has been developed within the Primary Care area of the Andalusian Public Health System. The venous blood sampling questionnaire was translated into and adapted to Spanish by qualified professionals and expert translators. The questionnaires were then delivered to all staff nurses from the health districts involved. The total sample consisted of 224 primary care nursing professionals. The factors that showed statistically significant relationships were identification and sample collection, management with information search, storage and labelling of samples, and reporting of errors. A lack of global relationship between factors makes it impossible to find a global quality factor in the sampling process. The process of translation, adaptation, and piloting of the questionnaire from its original version to the Spanish one has proven to be understandable by professionals in its entirety and to offer data similar to the original version.

## 1. Introduction

The preanalytical phase encompasses a set of processes that are difficult to define and delimit since they are developed in different places and at different times. Classically, it includes all processes from the time a physician makes a laboratory request until the sample is ready for testing. This is a vital part of the overall sampling process, as this is the time when more professionals from different disciplines will intervene: from the physician who makes the sampling request to the warden who transports the sample to the laboratory, as well as technical, administration, and nursing staff [1].

Of the total potentially detectable errors in the laboratory, preanalytical errors account for up to 70%. Specifically, distributions of 68.2% in the preanalytical phase [2,3,4], 13.3% in sampling, and 18.5% in the postanalytical phase [4], or 84.52%, 4.35%, and 11.13%, respectively [5], are observed. As can be seen, these figures show that most errors take place during the preanalytical phase, this being a matter of particular concern. In this sense, the role of nurses can be crucial as they are in charge of the identification, taking, and pre-processing of biological samples, mainly blood samples [1].

In our field, these errors are more likely to appear in greater numbers in samples coming from Primary Care (PC) [6], so developing procedures towards tackling this problem is considered a priority. Among other options, specific training activities have been designed and quantitative and qualitative analyses of the problem have been carried out [7,8,9,10,11].

The questionnaire “Regarding Blood-Sampling Practices at Primary Health Care Centres” was designed as a complementary tool for the techniques performed by healthcare professionals to measure deviations from the most appropriate practices during venous blood sampling [12]. It should be borne in mind that the use of professional-focused questionnaires is widespread in our work environment for the analysis of different technical and practical aspects [13,14]. However, this questionnaire has not been adapted to or validated in the Spanish language, so its use in our field as a complementary measure to the procedures carried out requires this previous adaptation.

Therefore, this study aims to carry out a transcultural adaptation and piloting of the “REGARDING BLOOD-SAMPLING PRACTICES AT PRIMARY HEALTH CARE CENTRES” questionnaire in Primary Care blood sampling.

## 2. Materials and Methods 

### 2.1. Design

A cross-sectional descriptive study, questionnaire validation, and a mixed multidisciplinary multicentre (quantitative-qualitative) study were developed in the Primary Care area of the Andalusian Public Health System (SSPA, for its acronym in Spanish) of the province of Huelva.

### 2.2. Participants

Professionals from the Huelva Costa and Condado Campiña districts of Huelva (Spain) participated in the study. 

A total of 224 PC nursing professionals that performed venous blood sampling, at least as a monthly practice, were included. A simple random sampling was carried out.

### 2.3. Instruments 

The VBSQ (venous blood sampling questionnaire) is an instrument designed at the Clinical Chemistry Laboratory (Umea University Hospital) and at the Department of Nursing and Department of Medical Biosciences (Umea University) to measure deviations from the best practices regarding venous blood sampling [12].

The development of the VBSQ was based on the international recommendations for venous blood sampling, thus designing a Swedish version that was subjected to validity of content analysis, apparent validity, and reliability analyses with a sample of professionals from the Swedish Health Care Service. The questionnaire was designed to be easy to read, with a reasonable number of items to ensure that the questionnaire could be completed within a reasonable period of time [12].

The VBSQ includes general characteristics of the healthcare professional (6 items), patient identification and sample collection (4 and 10 items, respectively), sample storage and information search (2 and 7 items), management of test requests and labelling of the sample (4 and 12 items), and error report frequency and suggestions (3 and 8 items). Most items were designed based on a Likert scale with a four-point semantic differential (never, rarely, often, and always).

### 2.4. Validation and Transcultural Adaptation

The VBSQ questionnaire was published in English. The formulation of the questions followed the same scheme as the one in the VBSQ. For its use in the Spanish context, the questionnaire was translated into and adapted to Spanish by qualified professionals and expert translators so as to reduce possible modifications with respect to the original scale and to keep its properties when used in other languages. For this cultural adaptation, the questionnaire was triangulated by two bilingual Spanish-English professors from the School of Nursing at the University of East Anglia (UK) and Huelva (Spain).

### 2.5. Procedure

The questionnaires were delivered on paper to all staff nurses in the involved health districts and through those responsible for their units by e-mail (see Table S1: Questionnaire Spanish).

### 2.6. Statistical Analysis

After the translation and transcultural adaptation of the questionnaire, its piloting process was carried out with the aim of assessing its internal consistency. To do this, the items were processed with the Cronbach’s test. The data set has 27 observations (questions) and the Cronbach’s alpha of the chosen items was 0.7194. Subsequently, the data obtained were descriptively analysed, using central trend and dispersion measures for those variables that allowed their usage and using frequency distribution for the qualitative ones. A multivariate analysis was also carried out to assess the possible associations between the different variables.

First, a univariate analysis was performed for the sample description (sex, age, seniority, work centre...) and the various quality indicators of the blood sampling process, based on frequency tables and valid percentages.

Second, an analysis of the main components was performed to determine the structure of each questionnaire dimension, analysing the standardised factorial loads of each item regarding the factor under which these were collected. For the factors’ construction, the relevance of each item was determined using 0.3 as the cut-off point in the factorial load of the component, unless the preservation of that item was relevant from a theoretical point of view.

Subsequently, for the identification of possible patterns in the practices of professionals during blood sampling, a two-stage cluster analysis was performed, where each of the variables related to this process were included. From the analysis itself, the number of groups in which the sample total could be segmented was determined. This analysis automatically determined the groups or clusters each subject belonged to, based on their responses to each of the indicators that formed the questionnaire. The cluster variable defining belonging was then crossed with each of the variables included in the analysis and with the different factors obtained in the previous step, thus defining each of the groups.

In this bivariate analysis, a proportion contrast test based on the Chi-squared test (χ²) was first used, together with the interpretation of the corrected standardised residuals (SRij > |1.96|) to avoid the possibility of not detecting differences due to the sample size (type II error).

Similarly, for the crossing of the cluster groups and the various factors obtained through the factorial analysis, the Mann-Whitney U test was used to compare means (because normality was not accepted in most variables), normality having been proven through the Kolmogorov-Smirnoff test.

Although certain factors met the conditions for applying parametric tests (normality and homoscedasticity), in this section, non-parametric tests were chosen in all cases to simplify the presentation of results, thus correcting the possible conservative effect of such tests by applying the corresponding effect size tests.

Finally, the relationship of each group was contrasted with different professionals’ characteristics (seniority, years of training, etc.) using both the contrasts of proportions and the contrasts of means, all of which were described for the previous section.

In all cases, a statistical significance of 5% was required (*p* < 0.05) by performing statistical analyses through the IBM SPSS Statistics statistical programme, version 22.0, licensed by the University of Huelva, with the support of extra calculation and layout add-ins provided by the Microsoft Excel programme.

### 2.7. Ethical Aspects

Subjects’ participation has been completely voluntary, guaranteeing anonymity at all times. Likewise, the participants signed an informed consent in which they were provided with information about the study, following the regulations in force (Organic Law 3/2018, of 5 December, on the Protection of Personal Data and guarantee of digital rights). The law of patient autonomy was not followed since the participants were workers of the health system.

## 3. Results

The total sample consisted of 224 PC nursing professionals, with professional activity in which venous blood sampling was, at least, a monthly practice (48% performed it daily and 43% at least once a week). In the study area, the budgeted staff is formed by 323 nurses. With a 95% confidence level and an estimated error of 5%, the representative sample was constituted by 176 subjects. So as to avoid losses, the sample size was overestimated by 50%, delivering 264 questionnaires. The response rate was 84.84%.

As for the work centre, 125 professionals belonged to the different Clinical Management Units (CMU) of the Province of Huelva, while 13 were pending assignment and the rest (86) belonged to several other CMUs not affiliated to the Andalusian Public Health System in Huelva. Of the sample of professionals who belonged to Huelva CMUs, 71% worked in a unit with students in training and 46.5% in an accredited unit. The average seniority in the CMUs was of around 10 years, although with great variability (mean = 9.97, standard deviation = 9.31).

*Preliminary analysis of the internal structure of the questionnaire on the practice of blood sampling in Primary Care facilities.*

- Sample Identification Factor

The first set of analysed items is a single factor composed of three items, two of them with positive charge and a third one with negative charge. All of them have a load greater than 0.3 in absolute value (see Table 1).

- Sample Collection Factor

For this set, a single factor was formed, composed of three items, all of them with positive charge and with a load greater than 0.6 in absolute value. For the design of this factor, the scores of the first two items have been reversed (see Table 1).

- Information Search Factor

The third set of items consists of four items joined into a single factor and with a positive load greater than 0.3 in absolute value (see Table 1).

- Sample Storage Factor

For this set, a single factor was formed, composed of three items, all with positive charge and with a load greater than 0.5, after reversing the scores of the first two items (see Table 1).

- Request Management Factor

The fifth set of items consists of four items joined in a single factor, with a positive load greater than 0.3, but with a high difference between the factorial loads (see Table 2).

- Analytical Tube Labelling Factor

For this set, a single factor was formed, consisting of five items, all with positive charge, after reversing the scores of the first, second, and fourth items. In addition, a low factorial load of items two and five is present, bringing little load to the overall factor (see Table 2).

- Error Reporting Factor

The seventh set of analysed items forms a single factor, composed of four items, all reversed and with a factorial load greater than 0.5 (see Table 2).

- Suggestions Factor

The last set of items consists of two items joined into a single factor, with a positive load greater than 0.7 (see Table 2).

- Correlation between Factors

As shown in Table 3, the factors do not generally present any high significant correlation between them, with statistically significant relationships between searching for information with identification (r = 0.147) and sample collection (r = 0.138), request management with information search (r = 0.230) and storage (r = 0.242) and labelling (r = 0.185) of samples, storage and reporting of errors (r = 0.216), and suggestions with sample identification (r = 0.227).

This lack of global relationship between factors makes it impossible to find a global quality factor in the sampling process, suggesting a differentiated analysis for each of the factors.

## 4. Discussion

As previously mentioned, the study of preanalytical errors and their origin and possible prevention has become a research area that has been offering results in recent years [6,7,8,9,10,11]. One of the most relevant observed aspects is that knowledge about the procedures associated with the nursing practice in PC is more directly related to the preanalytical phase: sampling, especially blood sampling.

In this sense, in general, the questionnaire worked well for the analysed Spanish nursing population when evaluating the usual venous blood sampling practices. The process of translation, adaptation, and piloting of the questionnaire from its original version to the Spanish one has proven to be fully understandable by professionals and to offer similar data to the original version [12], with no major problems. This is thanks to the development of expert testing, triangulation with natives, bilingual clinical staff and teaching professionals, and their piloting of the process. However, it is noteworthy that, after its implementation and analysis, an error was detected in the original English version of the questionnaire. After retrieving the original thesis, the translation of the Swedish version into English contained errors. Specifically, the term “college” appeared when in fact it was supposed to be “colleague”. This error was reported to the Swedish research team and led both groups to establish ties which have allowed them to design future joint studies [15].

A relevant aspect of this study was the high response rate (84.84%), which provided a considerably higher sample than expected. This was due to the fact that the study was carried out based on a 50% estimation given the possibility of obtaining scarce participation.

Descriptive data from professionals have shown, in general, good indicators in the sampling practice for each of the aspects assessed through the questionnaire. However, some aspects that could be improved have been marked (although the percentages of malpractice are significantly lower than those of good practices in all indicators). This need for improvement had already been found in the literature [16].

The analysis of the possible unidimensionality of each factor through the questionnaire has shown good results, although these must be confirmed with a greater number of participants and in other scenarios so that a quantitative score for each of these factors can be obtained (see tables).

The use of cluster analysis for profiling has shown good results, obtaining markedly different groups in almost all the analysed factors and indicators, automatically determining two groups with high and low compliance, respectively.

Finally, the use of variables such as teaching and accreditation of the work centre, as well as age, seniority, frequency in sampling, and training in this field have marked how each of these variables influence different factors in the sampling process. These associations, while weak, mark the need to expand the information and study the related factors the proper realization of the venous blood sampling process. Such investigations can be based on this questionnaire, taking it as a starting point for the assessment of the quality of this process.

While quality control systems designed to ensure the quality of the analytical phase are highly developed and in use in most clinical laboratories, this is not as prevalent in the preanalytical phase, even though it is considered as a possible threat for the process [7] and that accreditation procedures were described years ago [17].

One reason may be that laboratory professionals might have considered the analytical phase as the most important process in their profession, paying less attention to the preanalytical one. Outsourcing (in some environments) of the sampling process could be another cause, even though it was considered as a quality criterion and is now conceived as a key aspect [17,18]. Both aspects could have resulted in a situation of possible lack of coordination that may be highlighted by an increase in preanalytical errors, possibly requiring better implementation of quality assurance systems. Many of these aspects have already been declared by participants in the preanalytical process [9]. To do this, it may be highly useful to find out the degree of knowledge about the implementation of the laboratory process in our environment.

## 5. Conclusions

The objective of the study was met with the questionnaire piloting and implementation, after prior adaptation to the expected healthcare context. In fact, the degree of acceptance should be highlighted here, as can be understood from the high response rate identified.

It is intended to further investigate this line of research by developing, among other activities, an analysis that includes Spanish-speaking professionals from South America to internationalise the use of the questionnaire, as well as an assessment of the possible implementation of an exchange programme that would allow a transcultural adaptation to English so as to increase the dissemination of the study.

## Figures and Tables

**Table 1:** Standardised factorial load of “Sample Identification”, “Sample Collection”, “Information Search”, and “Sample Storage”. (Source: self-prepared).

Sample Identification	Factorial Load
How and how often is the patient’s identity confirmed during blood sampling? [I ask the patient for his/her name and social security number]	0.315
How and how often is the patient’s identity confirmed during blood sampling? [I know the patient and I don’t confirm his/her identity]	−0.829
How and how often is the patient’s identity confirmed during blood sampling? [I confirm the patient’s identity through his/her id photo]	0.597
**Sample Collection**	
If a tourniquet is used during blood sampling, when is it removed? [before the first sampling]	0.648
If a tourniquet is used during blood sampling, when is it removed? [During the first sampling]	0.703
If a tourniquet is used during blood sampling, when is it removed? [When I’ve finished the sampling]	0.850
**Information Search**	
What do you do when unsure whether a sample has been taken properly? [I check for instructions (available at CAP) released by the lab]	0.659
What do you do when unsure whether a sample has been taken properly? [I check for instructions in the internal network main webpage]	0.825
What do you do when unsure whether a sample has been taken properly? [I ask the university]	0.608
What do you do when unsure whether a sample has been taken properly? [I phone the lab]	0.444
**Sample Storage**	
How do you store the tubes right after the sampling? [On the table or similar]	0.541
How do you store the tubes right after the sampling? [In the pocket of my uniform]	0.778
How do you store the tubes right after the sampling? [In a test-tube rack]	0.705

Note: standardised factorial load shown.

**Table 2:** Standardised factorial load of “Request Management”, “Tube Labelling”, “Error Reporting”, and “Suggestion Factors”. (Source: self-prepared).

Request Management	Factorial Load
How often do you perform the following tasks? [Comparing the patient’s name and social security number with the information contained in the test request]	0.631
How often do you perform the following tasks? [Using the test request somebody filled in]	0.547
Checking the test request, if somebody else had filled it in.	0.801
How often do you perform the following tasks? [Checking that the test request and the tube have the same identification (barcode) before sending the sample to the lab]	0.452
**Analytical Tube Labelling**	
When do you label the tube? [Before approaching the patient]	0.658
When do you label the tube? [With the patient present, right before the sampling]	0.104
When do you label the tube? [With the patient present, right after the sampling]	0.768
When do you label the tube? [Later]	0.711
When do you label the tube? [Somebody else labelled the tube]	0.201
**Error Report**	
If you didn’t report any error, what were the reasons? [I didn’t have time]	0.777
If you didn’t report any error, what were the reasons? [It wouldn’t make any difference]	0.579
If you didn’t report any error, what were the reasons? [Nobody does it]	0.682
If you didn’t report any error, what were the reasons? [It’s too difficult]	0.824
**Suggestions Factor**	
I have enough knowledge to develop my daily job regarding venous blood sampling and its management.	0.820
The correct collection and handling of venous blood samples is considered as a priority in my PCC	0.820

Note: standardised factorial load shown.

**Table 3:** Matrix of correlations between the different factors of the VBSQ scale. (Source: self-prepared).

	Sample Collection	Information Search	Sample Storage	Request Management	Tube Labelling	Error Report	Suggestions
Sample identification	−0.086	0.147 *	−0.035	0.103	0.065	−0.02	227 **
Sample collection	-	0.138 *	0.127	0.041	0.01	0.064	−0.049
Information search		-	−0.011	0.230 **	0.122	0.028	0.129
Sample storage			-	0.242 **	−0.002	0.216 **	0.044
Request management				-	−0.185 **	0.053	0.086
Tube labelling					-	0.138	0.041
Error report						-	−0.055

Note: marked with * *p* < 0.05 and ** *p* < 0.01.

## Supplementary Materials

The following are available online at https://www.mdpi.com/1660-4601/17/10/3541/s1, Table S1: Questionnaire Spanish.
