# Supplementary material for: Transcultural Adaptation and Piloting of the “Regarding Blood-Sampling Practices at Primary Health Care Centres” Questionnaire
Source: Int J Environ Res Public Health. 2020 May 19;17(10):3541. doi: 10.3390/ijerph17103541 (PMC7277440; doi:10.3390/ijerph17103541)
Supplement: Supplementary file 1 [file ijerph-17-03541-s001.pdf]

CUESTIONARIO EN RELACIÓN A LA PRÁCTICA DE TOMA DE MUESTRAS DE SANGRE EN LOS CENTROS DE ATENCIÓN PRIMARIA (CAP)

(Por favor, lea las instrucciones previas antes de proceder al cuestionario)

INSTRUCCIONES PARA COMPLETAR EL CUESTIONARIO.

Este cuestionario contiene preguntas relacionadas con la toma de muestras sanguíneas para la realización de análisis clínicos. En él, se le realizarán tanto preguntas del tipo Sí/No, como de respuesta múltiple en las que podrá contestar ***Nunca**, **Pocas veces**, **Frecuentemente** o **Siempre*** con respecto a un método concreto. Es importante seleccionar la alternativa más adecuada de las opciones ofrecidas: esto significa que deberá marcar sólo una respuesta entre las opciones. Se trata de un cuestionario validado en inglés que hemos adaptado a nuestro entorno. Su participación es anónima y totalmente voluntaria. Al final del cuestionario, solicitamos su opinión y sus sugerencias.

> ☒ *Seleccione sus respuestas marcando con una cruz en el interior de la casilla que considere más adecuada.*
> ■ *Si desea cambiar alguna respuesta, rellene completamente la casilla incorrecta.*

Nos interesa cómo se desenvuelve en su trabajo diario. Si no está seguro de cómo responder a una pregunta, responda lo que realiza de forma habitual.

En preguntas como la que aparecen en el ejemplo, es importante marcar una alternativa de cada fila.

Ejemplo de respuesta correcta:

1) ¿Qué utensilio usa para comer sopa?

		NUNCA	POCAS VECES	FRECUENTEMENTE	SIEMPRE
a)	Tenedor	☒	☐	☐	☐
b)	Cuchara	☐	☐	☐	☒
c)	Cuchillo	☒	☐	☐	☐

Una vez finalizado el cuestionario introdúzcalo en el sobre, ciérrelo y entréguelo en la dirección de su CAP. Recogeremos los sobres cuando finalice el período de la encuesta.

*Es crucial que responda las preguntas de forma honesta y con el mayor cuidado que sea posible. Es decir, debería **responder acerca de cómo actúa normalmente**, y no cómo se supone que debe hacerse.*

0 **Indíquenos por favor, su Centro de salud** o Consultorio: _ _ _ _ _ _ _ _ _ _ _ _ _ _ _ _ _

La siguiente sección contiene preguntas relacionadas con el año de nacimiento, la formación recibida y rutinas.

1) ¿En qué año nació?

19 _ _

2) ¿Durante cuánto tiempo lleva trabajando en éste CAP?

_ _ año/s _ _ mes/es

3) ¿Cada cuánto tiempo toma una muestra de sangre?

Cada día de trabajo ☐

Cada semana ☐

Cada mes ☐

Pocas veces ☐

Nunca ☐

4) ¿En qué año recibió la última formación sobre la toma de muestras sanguíneas?

_ _ _ _

5) ¿Con anterioridad a esa ocasión, en qué año recibió la formación sobre la toma de muestras sanguíneas?

_ _ _ _

6) ¿Le gustaría recibir educación acerca de la toma de muestras sanguíneas?

No ☐

Sí ☐

Las siguientes preguntas son referentes a la identificación del paciente y a la toma de la muestra.

Es importante que marque UNA casilla para cada fila del siguiente recuadro.

7) ¿Cómo y con qué frecuencia confirma la identidad del paciente cuando toma muestras de sangre?

	Nunca	Pocas veces	Frecuen temente	Siempre
a) Le pregunto al paciente su nombre +	☐	☐	☐	☐
b) Le pregunto al paciente su número de la seguridad social +	☐	☐	☐	☐
c) Le pregunto al paciente su nombre, y su número de la seguridad social ++	☐	☐	☐	☐
d) Conozco al paciente y no confirmo la identidad -	☐	☐	☐	☐
e) Confirmo la foto del documento de identidad del paciente -	☐	☐	☐	☐
f) Otras formas: (Indique cuales y frecuencia) _	☐	☐	☐	☐

8) Si utiliza ligadura o compresor durante la toma de muestra sanguínea, ¿cuándo lo quita?

	Nunca	Pocas veces	Frecuen temente	Siempre
a) Antes de tomar la primera muestra ++	☐	☐	☐	☐
b) Durante la toma de muestra +	☐	☐	☐	☐
c) Cuando he finalizado la toma de muestra -	☐	☐	☐	☐
d) Si hay problemas para tomar la muestra, mantengo la ligadura tanto tiempo como sea necesario. =	☐	☐	☐	☐

9) ¿Cuánto tiempo deja descansar al paciente (en decúbito supino o sentado) antes de la toma de muestra de sangre?

Nada	0-5 min	6-10 min	11-15 min	Más de 15 min	No lo compruebo
☐ -	☐ +	☐ ++	☐ +	☐ -	☐ -

10) ¿Cada cuánto tiempo realiza la siguiente tarea?

	Nunca	Pocas veces	Frecuentemente	Siempre
a) Si el tubo de ensayo tiene un aditivo, ¿invierte el tubo inmediatamente antes de que esté lleno?	☐	☐	☐	☐

Las siguientes preguntas están relacionadas con el almacenamiento y la búsqueda de información para la resolución de dudas clínicas.

Es importante que marque UNA casilla para cada hilera de la siguiente sección.

11) ¿Qué hace cuando no está seguro de que una muestra ha sido bien tomada?

	Nunca	Pocas veces	Frecuentemente	Siempre
a) Compruebo la lista de instrucciones (disponible en el CAP) emitida por el laboratorio. +	☐	☐	☐	☐
b) Compruebo la lista de instrucciones en la página principal de la red interna. +	☐	☐	☐	☐
c) Pregunto en la universidad -	☐	☐	☐	☐
d) Llamo al laboratorio +	☐	☐	☐	☐
e) Otros: (Indique cuales y frecuencia) _	☐	☐	☐	☐

12) ¿Cómo almacena los tubos inmediatamente después de tomar la muestra?

	Nunca	Pocas veces	Frecuentemente	Siempre
a) Sobre una mesa u otro sitio similar +	☐	☐	☐	☐
b) En el bolsillo de mi uniforme -	☐	☐	☐	☐
c) En un soporte para los tubos de analíticas ++	☐	☐	☐	☐
d) Otros: (Indique cuales y frecuencia) _	☐	☐	☐	☐

Las siguientes preguntas SÓLO se refieren a las solicitudes de pruebas.

Es importante que marque UNA casilla para cada hilera del siguiente recuadro.

13) ¿Normalmente indica usted la hora de la extracción en la petición?

Siempre	Casi siempre	Algunas veces	Pocas veces	Nunca
☐ ++	☐ +	☐ -	☐ -	☐ --

14) En caso de contestar afirmativamente (siempre o casi siempre) y de ser usted mismo quien anota la hora de la extracción, ¿en qué momento lo hace?

Nunca	Más de 30 minutos antes de la toma de muestra	0-30 min antes de la toma de muestra	0-30 min después de la toma de muestra	Más de 30 min después de la toma de muestra
☐ -	☐ -	☐ +	☐ +	☐ -

15) ¿Con qué frecuencia realiza las siguientes tareas? TODOS LOS NUNCA Y POCAS VECES (-),

	Nunca	Pocas veces	Frecuentemente +	Siempre ++
a) Comparar el nombre del paciente y el número de la seguridad social con la información de la petición de prueba.	☐	☐	☐	☐
b) Usar la solicitud de prueba que alguien ha rellenado	☐	☐	☐	☐
c) Firmar la petición de prueba	☐	☐	☐	☐
d) Comprobar la información de la petición de prueba, si alguien la ha completado	☐	☐	☐	☐
e) Comprobar que la petición de prueba y el tubo tienen la identificación (código de barras) antes de enviar la prueba al laboratorio.	☐	☐	☐	☐

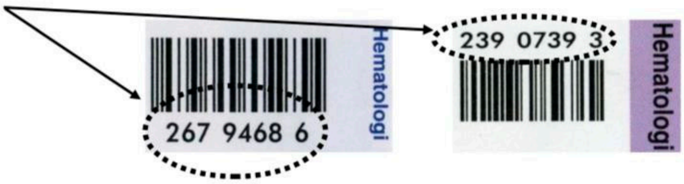

16) ¿Cuándo etiqueta el tubo?

	Nunca	Pocas veces	Frecuentemente	Siempre
a) Antes de acercarme al paciente	☐ +	☐ =	☐-	☐ -
b) Junto al paciente antes de tomar la muestra	☐ -	☐ -	☐ +	☐ ++
c) Junto al paciente después de tomar la muestra	☐ -	☐ -	☐ +	☐ ++
d) En una ocasión posterior	☐ ++	☐ +	☐ -	☐ -
e) Alguien ha etiquetado el tubo con antelación	☐ ++	☐ +	☐ -	☐ -
f) Otra persona etiqueta el tubo después de la toma de muestra	☐ ++	☐ +	☐ -	☐ -

---

Estas últimas preguntas están relacionadas con la notificación de errores y sugerencias.

Es importante que marque UNA casilla para cada fila del siguiente recuadro.

17) Aproximadamente, ¿cuántos errores ha notificado por escrito después de haber observado o cometido un error en una toma muestra de sangre venosa?

Número de veces	No he escrito ninguno
_ _ veces	☐

18) Si no has realizado esa notificación, ¿Cuál o cuáles fueron las razones?

(Por favor, responda las preguntas de abajo incluso si nunca ha escrito notificación)

TODAS SEERÍAN MALAS PRÁCTICAS	Nunca	Pocas veces	Frecuen temente	Siempre
a) No he tenido tiempo	☐ -	☐ -	☐ -	☐ -
b) No habría ninguna diferencia	☐ -	-	-	-
c) Nadie lo hace.	-☐	-	-	-
d) Es demasiado difícil	☐	☐	☐	☐
e) Otras razones: (Indique cuales y frecuencia) _	☐	☐	☐	☐

19) ¿En qué medida está de acuerdo con las siguientes afirmaciones?

	No demasiado de acuerdo	Bastante de acuerdo	Totalmente de acuerdo
a) Tengo el suficiente conocimiento para mi trabajo diario respecto a la toma de muestra de sangre venosa y su manipulación	☐-	+	++
b) La correcta recolección y manejo de las muestras de sangre venosa están consideradas una prioridad en mi CAP	☐-	+	++

20) **Por último, si hubiera cualquier cuestión o sugerencia que quisiera realizar, por favor hágalo** _ _ _ _
_ _
_ _
_ _
_ _

¡Gracias por cumplimentar el cuestionario!

Por favor, asegúrese de haber completado todas las preguntas. Después introduzca el cuestionario en el sobre anónimo, ciérrelo, y entréguelo en la dirección de su CAP
